# Synergistic Enhancement of Electrocatalytic Oxygen Evolution via Photothermal Effect in NiFeS/Cs_0.32_WO_3_

**DOI:** 10.3390/molecules31132330

**Published:** 2026-07-02

**Authors:** Ze Wang, Xin Zhang, Wucong Wang, Xiong Yang, Xinyu Song, Shifeng Wang

**Affiliations:** 1 Key Laboratory of Plateau Oxygen and Living Environment of Xizang Autonomous Region and College of Science, Xizang University, Lhasa 850000, Chinayangx@ustb.edu.cn (X.Y.);; 2 School of Energy and Environmental Engineering, University of Science and Technology Beijing, Beijing 100000, China

**Keywords:** oxygen evolution reaction, heterostructure, photothermal effect, NiFeS

## Abstract

Photothermal-assisted electrocatalysis is an effective approach to enhance the efficiency of the oxygen evolution reaction (OER), but the synergistic mechanism between the photothermal effect and the regulation of catalyst electronic structure remains unclear. This work reports the construction of NiFeS/Cs_0.32_WO_3_ heterostructures, which integrate interfacial electron transfer and localized surface plasmon resonance (LSPR)-induced photothermal effects to enhance OER performance. The Cs_0.32_WO_3_ component with hexagonal tungsten bronze structure exhibits strong absorption in the near-infrared region, attributed to LSPR (1100 nm to 2500 nm) and small polaron transition (780 nm to 1100 nm), endowing the NiFeS/Cs_0.32_WO_3_ composite with excellent photothermal conversion capability. Under 808 nm laser irradiation, the steady-state surface temperature of the heterostructure reaches 65.1 °C. X-ray photoelectron spectroscopy and ultraviolet photoelectron spectroscopy analyses reveal that spontaneous electron transfer from NiFeS to Cs_0.32_WO_3_ occurs at the heterostructure interface, thereby optimizing the electronic structure of active sites. Electrochemical measurements demonstrate that at a current density of 50 mA cm^−2^, the NiFeS/Cs_0.32_WO_3_ composite exhibits an overpotential of 301 mV under near-infrared irradiation, representing a reduction of 53 mV compared to NiFeS under dark conditions. At a current density of 50 mA cm^−2^, the photothermal enhancement effect of the NiFeS/Cs_0.32_WO_3_ composite is identified as the predominant contributor to the overall performance improvement. Nevertheless, the intrinsic interfacial effect associated with the heterojunction also plays a crucial role and makes a non-negligible contribution to the enhanced electrocatalytic activity. The Tafel slope decreases from 57.8 mV dec^−1^ to 44.5 mV dec^−1^ under near-infrared illumination, indicating accelerated OER kinetics. This work elucidates the mechanism of synergistic enhancement between heterostructure construction and photothermal effects, providing insights for the design of advanced photothermal electrocatalysts.

## 1. Introduction

The rapid depletion of fossil fuel reserves and the increasingly severe environmental deterioration caused by carbon emissions have driven global research into clean and renewable energy alternatives [1,2]. Hydrogen possesses an extremely high gravimetric energy density (approximately 120 MJ kg^−1^) and produces only water as a combustion product, making it a crucial energy carrier for achieving carbon neutrality [3,4]. Electrochemical water splitting driven by renewable electricity is one of the most promising approaches for sustainable hydrogen production, offering advantages such as high product purity, environmental friendliness, and scalability [5]. However, the large-scale application of this technology is still severely constrained by the sluggish intrinsic kinetics of the anodic oxygen evolution reaction (OER), which is a four-electron transfer process accompanied by the generation of multiple oxygen-containing intermediates (*OH, *O, *OOH) and substantial thermodynamic energy barriers [6,7]. Currently, iridium dioxide (IrO_2_) and ruthenium dioxide (RuO_2_), owing to their favorable electronic configurations and near-optimal binding energies with reaction intermediates, remain the benchmark electrocatalysts for OER [8]. Nevertheless, these noble metal oxides suffer from high cost, low crustal abundance, and poor long-term operational stability, severely limiting their practical application in large-scale commercial electrolyzers [9,10]. Therefore, developing earth-abundant transition metal-based electrocatalysts with competitive catalytic activity and durability has become an important research direction [11].

Among numerous candidate materials, nickel-iron-based compounds have attracted attention due to the strong synergistic interactions between Ni and Fe centers, where the incorporation of Fe can modulate the oxidation state of Ni and promote the formation of catalytically active oxyhydroxide species during OER [12]. Heterostructure construction for interfacial engineering has become an effective strategy to overcome the inherent limitations of single-component materials and enhance electrocatalytic performance [13,14]. At heterostructure interfaces, the contact between materials with different work functions spontaneously induces electron redistribution and forms a built-in electric field, which can effectively modulate the local electronic environment of catalytically active sites [15]. Interfacial charge transfer phenomena can optimize the d-band center position of transition metals, regulate the adsorption energies of OER intermediates, and reduce charge transfer resistance [16,17]. To date, numerous heterostructure architectures have been constructed to promote OER electrocatalysis, including Mott–Schottky contacts (Ru-RuO_2_, Co/CoP) [13,17], semiconductor–semiconductor junctions (NiS/MoS_2_, NiFe-LDH/NiS, Ni_3_Se_2_/NiSe) [18,19,20], and carbon-supported composite structures (Ni/CeO_2_@N-CNFs, WN-Ni@N,P-CNT, Co-Mo_2_C/CNx) [21,22,23].

Beyond the modification of catalysts themselves, the introduction of external energy fields represents a novel approach to break through the limitations of conventional catalytic performance [24]. Among various external stimuli, photothermal-assisted electrocatalysis has attracted extensive research as an energy-efficient enhancement strategy [25,26]. Light-absorbing materials generate localized surface heating upon irradiation, which thermodynamically reduces the required overpotential according to the Nernst equation and kinetically accelerates mass transfer and charge transfer processes [27,28]. Additionally, the localized heating effect facilitates the detachment of evolved gas bubbles from the electrode surface, maintaining effective accessibility to active sites during continuous operation [29]. Photothermal enhancement strategies have been successfully applied to numerous materials including nickel sulfides [29], nickel phosphides [30], cobalt-based compounds [31], MXenes [32], and plasmonic metal nanostructures [33], capable of operating under solar or near-infrared (NIR) irradiation [34,35,36,37]. Cesium tungsten bronze (Cs_0.32_WO_3_) is a plasmonic semiconductor with exceptional near-infrared absorption properties [38]. The hexagonal tungsten bronze structure of Cs_0.32_WO_3_ features hexagonal tunnel channels that accommodate Cs^+^ ions, which act as electron donors to generate abundant free carriers, producing localized surface plasmon resonance (LSPR) absorption in the range of 1100 to 2500 nm [38,39]. Furthermore, small polaron transitions between adjacent W^5+^ and W^6+^sites contribute additional absorption in the spectral region from 780 nm to 1100 nm [39]. The dual absorption mechanisms endow Cs_0.32_WO_3_ with excellent photothermal conversion efficiency under near-infrared irradiation.

This work reports the rational design and synthesis of NiFeS/Cs_0.32_WO_3_ heterostructures, which integrate heterostructure-mediated electronic modulation with LSPR-enhanced photothermal effects to achieve efficient OER electrocatalysis. The composite catalyst was prepared via a facile two-step solvothermal method, and interfacial electron transfer from NiFeS to Cs_0.32_WO_3_ was elucidated using X-ray photoelectron spectroscopy (XPS) and ultraviolet photoelectron spectroscopy (UPS) analyses. UV-vis-NIR diffuse reflectance spectroscopy and infrared thermal imaging demonstrated that the heterostructure possesses excellent photothermal conversion performance, achieving a steady-state temperature of 65.1 °C under near-infrared irradiation. Importantly, quantitative decoupling analysis was employed to separately determine the contributions of heterostructure effects and photothermal effects to the overall OER enhancement. To place the present work in a broader academic context, the photothermally enhanced OER performance of the NiFeS/Cs_0.32_WO_3_ composite was systematically compared with recently reported state-of-the-art systems, including NiFe-LDH/plasmonic metal composites [40] and transition metal sulfide/oxide heterostructures [41]. Compared with NiFe-LDH-based system, the present catalyst exhibits a low Tafel slope of 44.5 mV dec^−1^. Moreover, the incorporation of NiFeS endows the catalyst with superior intrinsic electrical conductivity, thereby facilitating efficient charge transfer during the OER process. In comparison with transition metal sulfide/oxide heterostructures, the proposed NiFeS/Cs_0.32_WO_3_ architecture features a simpler structural design while achieving competitive photothermal enhancement, thus avoiding the complexity associated with multi-heterojunction interface engineering. This work provides mechanistic insights and design strategies for the development of advanced photothermal electrocatalysts.

## 2. Results and Discussion

**Figure 1:**
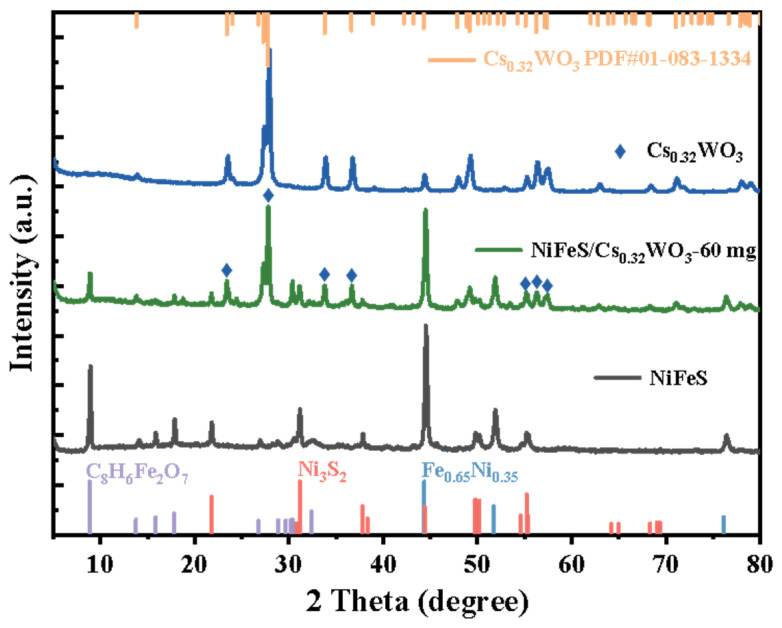
XRD patterns of Cs_0.32_WO_3_, NiFeS, and NiFeS/Cs_0.32_WO_3_-60 mg composite.

X-ray diffraction (XRD) was employed to analyze the crystal structure and phase composition of the prepared samples. Figure 1 shows the XRD patterns of Cs_0.32_WO_3_, NiFeS, and NiFeS/Cs_0.32_WO_3_-60 mg composite. The diffraction pattern of NiFeS shows that its diffraction peaks match well with the standard cards of C_8_H_6_Fe_2_O_7_ (PDF#00-033-1724), Fe_0.65_Ni_0.35_ (PDF#04-002-8942), and Ni_3_S_2_ (PDF#97-003-6338), indicating that NiFeS is a multiphase composite material with successful incorporation of metal sulfides. Among these phases, Ni_3_S_2_ belongs to the rhombohedral crystal system with lattice parameters of a = b = c = 4.08 Å and α = β = γ = 89.42°. The characteristic diffraction peaks appearing at 2θ = 21.8°, 31.1°, 37.8°, 44.5°, and 54.8° correspond to the (100), ($\bar{1}10$), (111), (200), and (211) crystal planes of Ni_3_S_2_, respectively. The formation of the C_8_H_6_Fe_2_O_7_ phase is attributed to the coordination interaction between terephthalic acid and iron ions during the solvothermal process, while the formation of the Fe_0.65_Ni_0.35_ alloy phase results from the partial reduction of Fe^3+^ and Ni^2+^ under reducing atmosphere. The diffraction pattern of Cs_0.32_WO_3_ is in excellent agreement with the standard card PDF#01-083-1334, exhibiting a typical hexagonal tungsten bronze structure (a = b = 7.4116 Å, c = 7.5981 Å, α = β = 90°, γ = 120°). The characteristic peaks at 23.5°, 27.4°, 27.9°, 36.7°, and 49.3° belong to the (002), (102), (200), (202), and (220) crystal planes, respectively. The sharp and intense diffraction peaks indicate that the prepared Cs_0.32_WO_3_ possesses high crystallinity. In the diffraction pattern of the NiFeS/Cs_0.32_WO_3_ composite, in addition to retaining all the characteristic diffraction peaks of NiFeS, the characteristic diffraction peaks of Cs_0.32_WO_3_ are clearly observed at 2θ = 23.4°, 27.3°, 27.8°, 33.8°, and 36.7°, confirming the successful compositing of the two materials. It is noteworthy that the diffraction peaks of Cs_0.32_WO_3_ in the composite are slightly shifted to lower angles compared to the pure phase sample, which may be attributed to the slight lattice expansion of Cs_0.32_WO_3_ caused by interfacial interactions between NiFeS and Cs_0.32_WO_3_.

**Figure 2:**
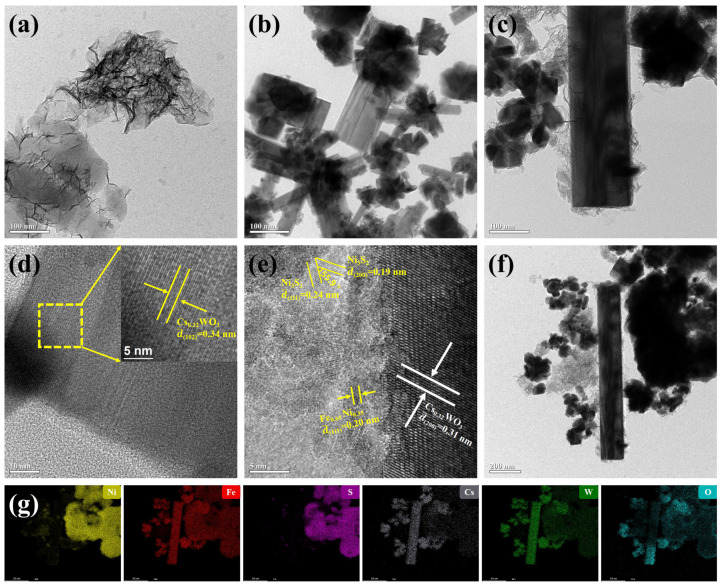
(**a**) TEM image of NiFeS; (**b**,**d**) TEM and HRTEM images of Cs_0.32_WO_3_; (**c**,**f**) TEM images and (**e**) HRTEM images of NiFeS/Cs_0.32_WO_3_-60 mg composite; (**g**) EDS elemental mapping images of NiFeS/Cs_0.32_WO_3_-60 mg composite.

Transmission electron microscopy (TEM) was employed to characterize the morphology and microstructure of the prepared samples. As shown in Figure 2a, NiFeS exhibits a flower-like structure assembled from ultrathin nanosheets, which are interwoven and stacked to form an abundant porous network structure that is beneficial for increasing the specific surface area of the material and providing more active sites. Figure 2b shows the TEM image of Cs_0.32_WO_3_, revealing that Cs_0.32_WO_3_ exhibits a regular polyhedral morphology with well-defined edges, indicating good crystallinity. Figure 2c,f present TEM images of the NiFeS/Cs_0.3_2WO_3_-60 mg composite, showing that Cs_0.32_WO_3_ polyhedral particles are distributed within the NiFeS nanosheet network, forming intimate contact interfaces between the two phases, which is favorable for rapid charge transfer at the interface. To further elucidate the crystal structure of the samples, high-resolution transmission electron microscopy (HRTEM) analysis was conducted. Figure 2d shows the HRTEM image of Cs_0.32_WO_3_, where the measured lattice spacing is 0.34 nm, corresponding to the (102) crystal plane of Cs_0.32_WO_3_. Figure 2e displays the HRTEM image of the NiFeS/Cs_0.32_WO_3_ composite, where lattice fringes of different phases can be observed within the same field of view. Among them, the lattice fringes with spacings of 0.24 nm and 0.19 nm correspond to the (111) and (200) crystal planes of Ni_3_S_2_, respectively, with a measured interplanar angle of 55.9°, which is very close to the theoretical value of 55.2°, further confirming the presence of the Ni_3_S_2_ phase. Additionally, the lattice fringe with a spacing of 0.31 nm is attributed to the (200) crystal plane of Cs_0.32_WO_3_, which is slightly smaller than the standard d-spacing of pure Cs_0.32_WO_3_ (0.32 nm), echoing the observation from XRD analysis of diffraction peak shifts to lower angles and slight lattice expansion in the composite, indicating interfacial interactions between NiFeS and Cs_0.32_WO_3_. The above HRTEM results corroborate the XRD analysis, fully confirming the multiphase coexistence characteristics in the composite material. Figure 2g shows the energy-dispersive X-ray spectroscopy (EDS) elemental mapping images of the NiFeS/Cs_0.32_WO_3_ composite. The results reveal that Ni, Fe, and S elements are predominantly distributed in the nanosheet regions, while Cs, W, and O elements are mainly concentrated in the polyhedral particle regions, consistent with the morphological features observed by TEM. EDS line scanning results (Figure S1) further reveal the spatial distribution patterns of each element, showing that Ni and Fe signals exhibit distinctly complementary distribution characteristics with Cs and W signals, confirming the successful construction of the NiFeS/Cs_0.32_WO_3_ heterostructure.

**Figure 3:**
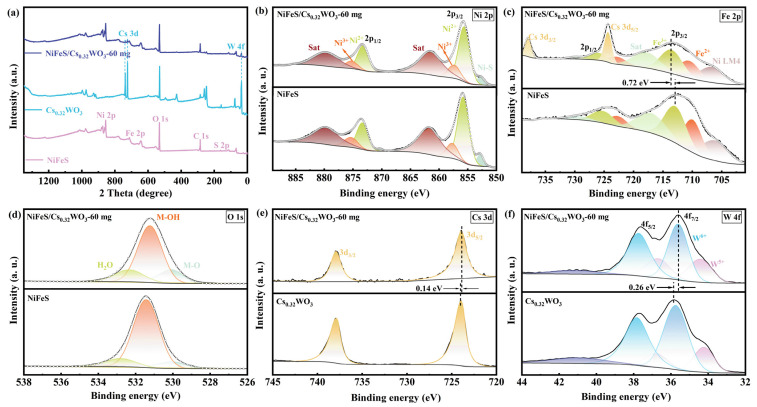
XPS spectra of NiFeS, Cs_0.32_WO_3_, and NiFeS/Cs_0.32_WO_3_-60 mg: (**a**) survey spectra; (**b**) Ni 2p; (**c**) Fe 2p; (**d**) O 1s; (**e**) Cs 3d; (**f**) W 4f.

The elemental composition and chemical states of the samples were characterized by X-ray photoelectron spectroscopy (XPS) (Figure 3). Figure 3a shows the XPS survey spectra of NiFeS, Cs_0.32_WO_3_, and NiFeS/Cs_0.32_WO_3_-60 mg composite. Characteristic peaks of Ni, Fe, S, O, and C elements were detected in the NiFeS sample, while the Cs_0.32_WO_3_ sample exhibited characteristic signals of Cs, W, and O elements. In the survey spectrum of the NiFeS/Cs_0.32_WO_3_-60 mg composite, characteristic peaks of Ni 2p, Fe 2p, S 2p, Cs 3d, W 4f, and O 1s were simultaneously observed, confirming the successful compositing of the two materials, consistent with XRD and EDS analysis results. To further elucidate the chemical states of each element and interfacial electronic interactions, high-resolution XPS analysis was performed on the main elements. As shown in Figure 3b, in the Ni 2p spectrum of the NiFeS sample, the peaks located at 855.8 and 873.4 eV correspond to the 2p^3/2^ and 2p^1/2^ orbitals of Ni^2+^, respectively, while the peaks at 857.7 and 875.3 eV are attributed to Ni^3+^, accompanied by satellite peaks (Sat.). Additionally, the characteristic peak of the Ni-S bond can be observed at 852.8 eV, indicating the presence of nickel sulfide. In the Fe 2p spectrum (Figure 3c), the peaks located at 712.9 and 725.3 eV in the NiFeS sample correspond to the 2p^3/2^ and 2p^1/2^ orbitals of Fe^3+^, respectively, while the peaks at 709.9 and 722.7 eV are attributed to Fe^2+^. In comparison, in the NiFeS/Cs_0.32_WO_3_ composite, the Fe 2p^3/2^ peak shifts toward higher binding energy by 0.72 eV, while this region also overlaps with the Cs 3d^3/2^ and Cs 3d^5/2^ signal peaks. The positive shift in Fe 2p binding energy indicates a decrease in electron cloud density around Fe, signifying electron transfer from NiFeS to Cs_0.32_WO_3_. The analysis results of the O 1s spectrum are shown in Figure 3d. The O 1s spectrum of NiFeS can be fitted into three peaks: the peak at 530.1 eV corresponds to metal–oxygen bonds (M-O), the main peak at 531.4 eV is attributed to metal–hydroxyl bonds (M-OH), and the peak at 533.8 eV corresponds to adsorbed water (H_2_O). For the Cs 3d spectrum (Figure 3e), the peaks located at 723.9 and 737.9 eV in the NiFeS/Cs_0.32_WO_3_ composite correspond to the 3d^5/2^ and 3d^3/2^ orbitals of Cs 3d, respectively. Compared to the pure NiFeS sample, the Cs 3d orbital peak shifts toward lower binding energy by 0.14 eV, indicating an increase in electron cloud density around Cs, signifying that Cs_0.32_WO_3_ has received electrons from NiFeS. From the W 4f spectrum (Figure 3f), it can be seen that the W 4f spectrum of the NiFeS/Cs_0.32_WO_3_ composite is fitted into two sets of spin–orbit splitting peaks, where the peaks at 35.6 and 37.7 eV correspond to the 4f^7/2^ and 4f^5/2^ orbitals of W^6+^, while the peaks at 34.4 and 36.7 eV are attributed to W^5+^. The presence of W^5+^ results from the intercalation of Cs^+^ into the WO_3_ lattice, causing partial reduction of W^6+^, which is a typical characteristic of cesium tungsten bronze. Compared to the pure NiFeS sample, the W 4f peak in the composite shifts toward lower binding energy by 0.26 Ev, further confirming electron transfer from NiFeS to Cs_0.32_WO_3_. According to the above XPS analysis results, significant electron transfer occurs at the NiFeS/Cs_0.32_WO_3_ interface, with electrons transferring from NiFeS to Cs_0.32_WO_3_. This interfacial electronic interaction can modulate the electronic structure of the catalyst, optimize the adsorption energies of OER intermediates, and thereby enhance electrocatalytic activity.

**Figure 4:**
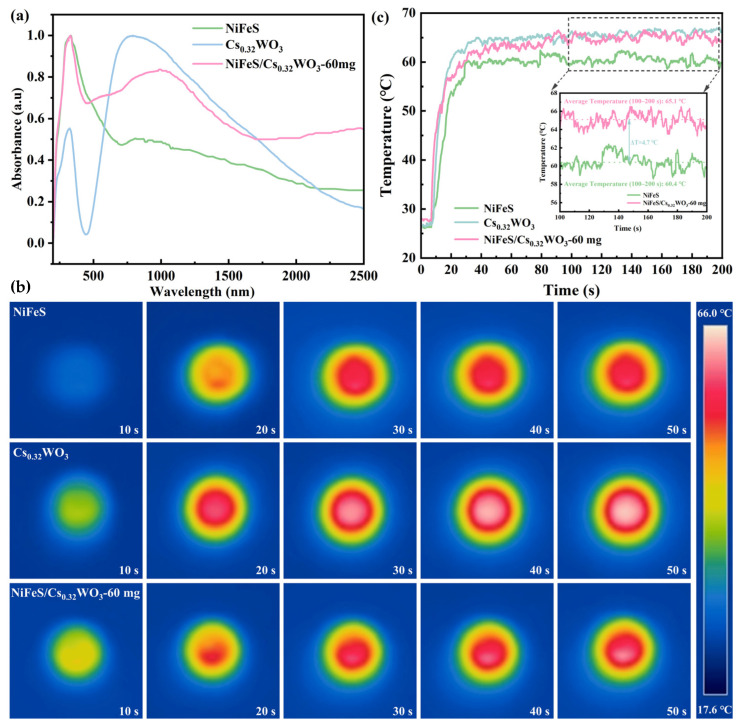
(**a**) UV-vis diffuse reflectance spectra (200–2500nm); (**b**) infrared thermal images of NiFeS, Cs_0.32_WO_3_ and NiFeS/Cs_0.32_WO_3_-60 mg under 808 nm laser irradiation; (**c**) temperature–time curves (inset: average temperature comparison during 100–200 s).

To investigate the effect of Cs_0.32_WO_3_ incorporation on the optical properties of the composite, the samples were characterized using UV–visible diffuse reflectance spectroscopy (UV-vis DRS). As shown in Figure 4a, NiFeS exhibits strong absorption in the ultraviolet region of 200–400 nm, but its absorption capacity decreases sharply in the visible and near-infrared regions. In comparison, the NiFeS/Cs_0.32_WO_3_-60 mg composite exhibits higher absorption intensity across the entire tested wavelength range, with particularly significant enhancement in the near-infrared region. This is attributed to the localized surface plasmon resonance (LSPR) effect of free electrons in the unique hexagonal tungsten bronze structure of Cs_0.32_WO_3_, which endows it with strong light absorption capability in the near-infrared region. To more intuitively evaluate the photothermal conversion performance of the samples, an 808 nm near-infrared laser was used to irradiate the samples, and the temperature changes in the materials were monitored in real-time using an infrared thermal imaging camera. Figure 4b shows the infrared thermal images of NiFeS, Cs_0.32_WO_3_, and NiFeS/Cs_0.32_WO_3_-60 mg composite under different laser irradiation times. From the thermal images, it can be observed that all three samples exhibit significant temperature rise under laser irradiation. Under the same irradiation time, Cs_0.32_WO_3_ and the NiFeS/Cs_0.32_WO_3_ composite demonstrate faster heating rates, with their thermal imaging regions showing orange-red color (representing higher temperatures) earlier, while the heating rate of NiFeS is relatively slower. As the irradiation time extends to 50 s, the central regions of both Cs_0.32_WO_3_ and the NiFeS/Cs_0.32_WO_3_ composite reach higher temperatures and display bright white color, while the central temperature of NiFeS is relatively lower, intuitively demonstrating that samples containing Cs_0.32_WO_3_ possess superior photothermal conversion capability. (The temperature data were obtained from surface temperature measurements of dry powder samples in air. These results are intended solely to qualitatively demonstrate the photothermal heating capability of the Cs_0.32_WO_3_ component and do not represent the actual temperature at the electrode/electrolyte interface under OER operating conditions.) Figure 4c shows the temperature versus time curves of the three samples under 808 nm laser irradiation. All samples exhibit rapid temperature increase at the onset of irradiation (0–40 s), followed by gradual approach to equilibrium. In terms of steady-state temperature, Cs_0.32_WO_3_ exhibits the most excellent photothermal conversion performance; the NiFeS/Cs_0.32_WO_3_-60 mg composite ranks second with an equilibrium temperature of approximately 65 °C; meanwhile, pure NiFeS has an equilibrium temperature of only about 60 °C. The inset shows a comparison of average temperatures during the 100–200 s period, where the average temperature of NiFeS is 60.4 °C, while that of the NiFeS/Cs_0.32_WO_3_-60 mg composite reaches 65.1 °C, with a temperature difference of approximately 4.7 °C. This indicates that the incorporation of Cs_0.32_WO_3_ enhances the photothermal conversion capability of the composite. From the above results, it can be seen that Cs_0.32_WO_3_, owing to its excellent near-infrared light absorption and photothermal conversion efficiency, can serve as an efficient photothermal component incorporated into the NiFeS system. Under illumination, the localized thermal effect generated in the composite can increase the electrolyte temperature, accelerate mass transfer processes at the electrode/electrolyte interface, reduce reaction activation energy, and thereby potentially achieve photothermal-assisted enhancement of electrocatalytic oxygen evolution performance.

**Figure 5:**
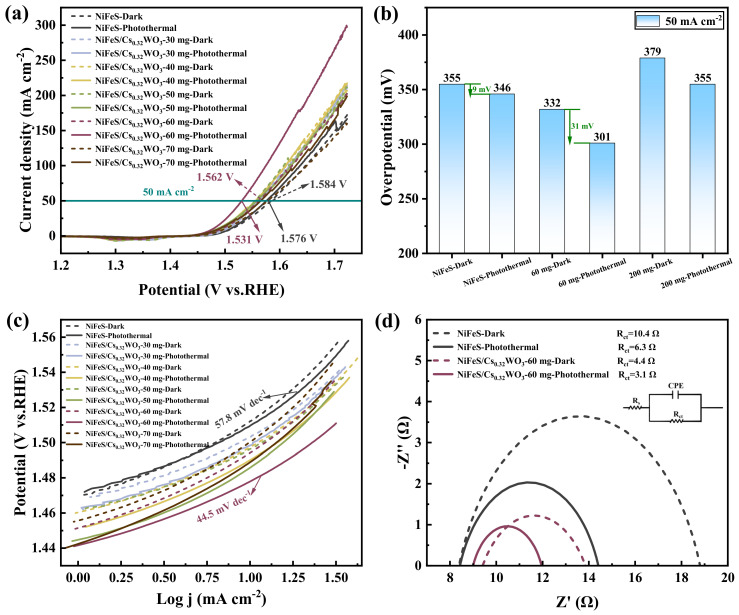
Electrocatalytic OER performance of NiFeS and NiFeS/Cs_0.32_WO_3_ composites under dark and photothermal conditions: (**a**) LSV curves; (**b**) overpotential comparison at 50 mA cm^−2^; (**c**) Tafel plots; (**d**) EIS plots (inset: equivalent circuit model).

To evaluate the electrocatalytic oxygen evolution reaction (OER) performance of the catalysts, tests were conducted in 1 M KOH electrolyte using a standard three-electrode system, and the catalytic activities under dark and photothermal conditions were compared. Figure 5a shows the linear sweep voltammetry (LSV) curves of NiFeS and NiFeS/Cs_0.32_WO_3_ composites with different Cs_0.32_WO_3_ additions under dark and photothermal conditions. It can be seen that the LSV curves of all samples shift toward lower potentials under photothermal conditions, indicating that the photothermal effect can promote OER kinetics. At the same current density, the NiFeS/Cs_0.32_WO_3_-60 mg composite exhibits the most excellent catalytic activity. To quantitatively analyze the respective contributions of heterostructure construction and photothermal effect to OER performance enhancement, systematic comparison of potentials under different conditions was conducted at a benchmark current density of 50 mA cm^−2^. As shown in Figure 5a, the potential required for NiFeS to reach 50 mA cm^−2^ under dark conditions is 1.584 V. After introducing Cs_0.32_WO_3_ to construct the heterostructure, the potential of the NiFeS/Cs_0.32_WO_3_-60 mg composite under dark conditions decreases to 1.562 V, a reduction of 22 mV, which can be attributed to the electron transfer effect at the heterostructure interface optimizing the electronic structure of the catalyst. Upon further introduction of the photothermal effect, the potential of the NiFeS/Cs_0.32_WO_3_-60 mg composite under photothermal conditions further decreases to 1.531 V vs. RHE, a reduction of 31 mV compared to dark conditions, which results from the localized thermal effect generated by the photothermal conversion of Cs_0.32_WO_3_. Therefore, the NiFeS/Cs_0.32_WO_3_-60 mg composite under photothermal conditions exhibits a potential reduction of 53 mV compared to NiFeS under dark conditions. The enhanced overall performance of the NiFeS/Cs_0.32_WO_3_ composite is primarily attributed to its photothermal enhancement effect, whereas the intrinsic interfacial effects arising from the heterojunction also contribute significantly and cannot be neglected. Quantitative decoupling analysis clearly demonstrates that heterostructure construction and photothermal assistance have synergistic enhancement effects on electrocatalytic performance improvement. As a control, the potential of NiFeS under photothermal conditions is 1.576 V, only 8 mV lower than under dark conditions, indicating that the photothermal response capability of pure NiFeS is limited. In contrast, the NiFeS/Cs_0.32_WO_3_-60 mg composite exhibits a potential reduction of 31 mV under photothermal conditions, which is 3.9 times that of NiFeS, demonstrating that Cs_0.32_WO_3_ is a crucial photothermal component for enhancing photothermal-assisted electrocatalytic performance. To more clearly compare the catalytic activities of various samples, Figure 5b shows the overpotentials of different catalysts at a current density of 50 mA cm^−2^. Under dark conditions, the overpotential of NiFeS is 355 mV, while that of the NiFeS/Cs_0.32_WO_3_-60 mg composite decreases to 332 mV. Upon introduction of the photothermal effect, the overpotential of NiFeS/Cs_0.32_WO_3_-60 mg further decreases to 301 mV. It is noteworthy that excessive Cs_0.32_WO_3_ loading (200 mg) actually decreases the catalytic activity, which is attributed to excessive Cs_0.32_WO_3_ covering the active sites of NiFeS, indicating the existence of an optimal composite ratio. Figure 5c shows the Tafel plots of various samples, used to evaluate OER kinetics. The Tafel slope of NiFeS under photothermal conditions is 57.8 mV dec^−1^, while that of the NiFeS/Cs_0.32_WO_3_-60 mg composite under photothermal conditions decreases to 44.5 mV dec^−1^. This indicates that the OER kinetics of the composite are faster under photothermal assistance. Figure 5d shows the Nyquist plots of NiFeS and NiFeS/Cs_0.32_WO_3_-60 mg composite under dark and photothermal conditions, with the inset showing the equivalent circuit model (Table S1). Fitting results show that the Rct values of NiFeS under dark and photothermal conditions are 10.4 Ω and 6.0 Ω, respectively, while those of the NiFeS/Cs_0.32_WO_3_-60 mg composite are 4.4 Ω and 3.0 Ω, respectively. From NiFeS under dark conditions (10.4 Ω) to NiFeS/Cs_0.32_WO_3_-60 mg under dark conditions (4.4 Ω), Rct decreases by 6.0 Ω, which is attributed to interfacial electron transfer at the heterostructure optimizing the charge transport pathway; from NiFeS/Cs_0.32_WO_3_-60 mg under dark conditions (4.4 Ω) to photothermal conditions (3.0 Ω), Rct further decreases by 1.4 Ω, which originates from the photothermal effect accelerating charge transfer at the electrode/electrolyte interface. Additionally, the double-layer capacitance (Cdl) of the samples at different scan rates (20–120 mV s^−1^) was determined by cyclic voltammetry to evaluate the electrochemical active surface area (ECSA) of the catalysts (Figure S2). As shown in Figure S2, the Cdl values of NiFeS, NiFeS/Cs_0.32_WO_3_-60 mg, and NiFeS/Cs_0.32_WO_3_-200 mg are 96.9, 309.1, and 211.6 μF cm^−2^, respectively. This indicates that the introduction of an appropriate amount of Cs_0.32_WO_3_ can increase the electrochemical active surface area of the catalyst, exposing more catalytically active sites. However, excessive Cs_0.32_WO_3_ doping (200 mg) causes a decrease in Cdl value, which is due to excessive Cs_0.32_WO_3_ accumulation covering some of the active sites of NiFeS, consistent with the OER performance analysis results.

**Figure 6:**
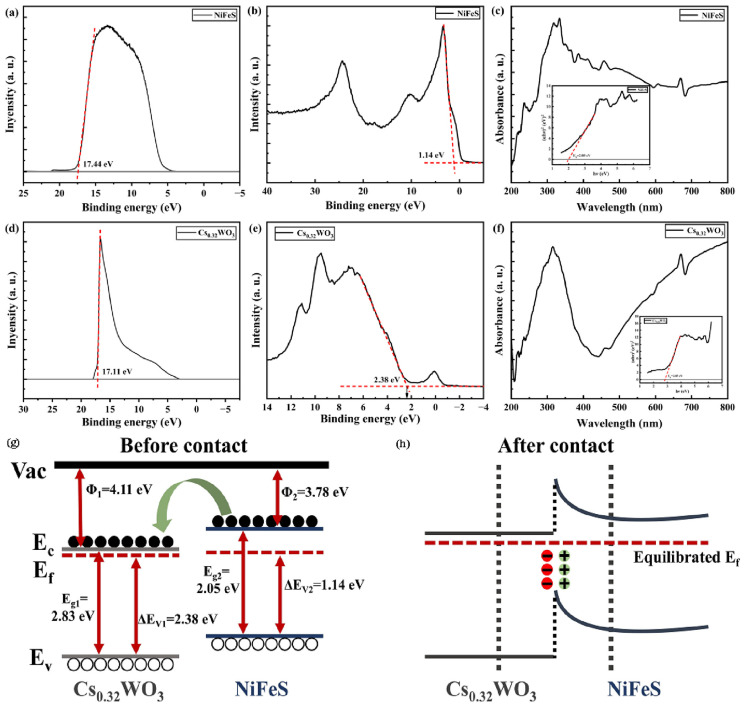
(**a**,**d**) UPS secondary electron cutoff spectra; (**b**,**e**) XPS valence band spectra; (**c**,**f**) UV-vis diffuse reflectance spectra (insets: Tauc plots); (**g**) schematic band structure before contact; (**h**) band structure and electron transfer after contact.

To further investigate the electron transfer mechanism of the NiFeS/Cs_0.32_WO_3_ heterostructure, UV-vis diffuse reflectance spectroscopy (DRS), ultraviolet photoelectron spectroscopy (UPS), and X-ray photoelectron spectroscopy valence band (XPS-VB) spectra were employed to determine the optical band gap (Eg), work function (Φ), and valence band edge (eV) of NiFeS and Cs_0.32_WO_3_, respectively. As shown in Figure 6a,d, the secondary electron cutoff (SEC) values of NiFeS and Cs_0.32_WO_3_ are 17.44 and 17.11 eV, respectively. According to the formula Φ = hν − SEC, with the excitation source radiation energy of 21.22 eV (hν = 21.22 eV), the calculated work functions are Φ_1_ = 4.11 eV for Cs_0.32_WO_3_ and Φ_2_ = 3.78 eV for NiFeS. As shown in Figure 6b,e, the positions of the valence band maximum relative to the Fermi level were determined by linear extrapolation of the XPS-VB spectra, with ΔEV values of 1.14 and 2.38 eV for NiFeS and Cs_0.32_WO_3_, respectively. As shown in Figure 6c,f, the band gaps are Eg1 = 2.83 eV for Cs_0.32_WO_3_ and Eg2 = 2.05 eV for NiFeS. Based on the above DRS, UPS, and XPS-VB analysis results, the energy band structure diagrams of NiFeS and Cs_0.32_WO_3_ were constructed (Figure 6g). Before contact, since the work function of Cs_0.32_WO_3_ (4.11 eV) is higher than that of NiFeS (3.78 eV), the Fermi level of Cs_0.32_WO_3_ is lower relative to the vacuum level. When the two materials come into contact to form a heterostructure (Figure 6h), due to the difference in Fermi levels, electrons will spontaneously flow from NiFeS with the higher Fermi level to Cs_0.32_WO_3_ with the lower Fermi level until equilibrium of their Fermi levels is achieved. The above energy band analysis results are mutually corroborated the XPS characterization results. This interfacial electron transfer effect optimizes the electronic structure of the catalyst, reducing the electron cloud density at NiFeS active sites, which is favorable for the adsorption and conversion of oxygen-containing intermediates (OH*, O*, OOH*) during the OER process, thereby enhancing electrocatalytic activity.

## 3. Materials and Methods

### 3.1. Materials

Terephthalic acid (PTA, 99.0%), nickel chloride hexahydrate (NiCl_2_·6H_2_O, analytical reagent, ≥98.0%), sodium sulfide (Na_2_S, ≥95.0%), N,N-dimethylformamide (DMF, ≥99.5%), tungsten hexachloride (WCl_6_, 99%), cesium hydroxide monohydrate (CsOH·H_2_O, analytical reagent), glacial acetic acid (CH_3_COOH, analytical reagent), and Nafion D-521 dispersion (5 wt%) were all purchased from Shanghai Aladdin Biochemical Technology Co., Ltd. (Shanghai, China). Iron(III) chloride hexahydrate (FeCl_3_·6H_2_O, analytical reagent) was purchased from Fuchen Chemical Reagent Co., Ltd. (Tianjin, China). Anhydrous ethanol (C_2_H_5_OH, analytical reagent) was supplied by Chengdu Jinshan Chemical Reagent Co., Ltd. (Chengdu, China). All chemical reagents were of analytical grade and used without further purification. Deionized water with a resistivity of 18.25 MΩ·cm, prepared using a Youpu ultrapure water system (UPR-II-10TNZ, Sichuan, China), was used throughout all experiments.

### 3.2. Preparation of Cs_0.32_WO_3_

First, 0.18 g of CsOH·H_2_O was dissolved in 32 mL of anhydrous ethanol under magnetic stirring at room temperature until complete dissolution, followed by the addition of 1.28 g of WCl_6_ with continued stirring until the solution became clear. Under continuous stirring, 8 mL of glacial acetic acid was slowly added dropwise and mixed thoroughly. The resulting precursor solution was transferred into a 100 mL Teflon-lined autoclave, which was then sealed and placed in an oven for hydrothermal treatment at 230 °C for 24 h. After the autoclave was naturally cooled to room temperature, the product was collected and washed alternately with deionized water and anhydrous ethanol three times by centrifugation, followed by vacuum drying at 60 °C for 12 h to obtain Cs_0.32_WO_3_ powder.

### 3.3. Preparation of NiFeS

First, a solvent system was prepared by mixing 32 mL of DMF, 2 mL of anhydrous ethanol, and 2 mL of deionized water. Subsequently, 1.6 mmol of PTA, 2.4 mmol of NiCl_2_·6H_2_O, 0.6 mmol of FeCl_3_·6H_2_O, and 0.6 mmol of Na_2_S were added to the above mixed solvent and subjected to ultrasonication for 30 min to achieve thorough dispersion. The obtained precursor solution was transferred into a 100 mL Teflon-lined autoclave, which was then sealed and maintained at 200 °C for 10 h. After the reaction was completed, the autoclave was naturally cooled to room temperature, the precipitate was collected and washed alternately with deionized water and anhydrous ethanol three times by centrifugation. The final product was dried in a vacuum oven at 60 °C for 12 h to obtain NiFeS powder samples.

### 3.4. Preparation of NiFeS/Cs_0.32_WO_3_

The preparation process of NiFeS/Cs_0.32_WO_3_ composite was similar to that of NiFeS, except that a certain amount of pre-synthesized Cs_0.32_WO_3_ powder was added to the precursor solution. After ultrasonication for 30 min, the mixture was transferred into a 100 mL Teflon-lined autoclave and maintained at 200 °C for 10 h. After natural cooling to room temperature, the product was washed alternately with deionized water and anhydrous ethanol three times by centrifugation, followed by vacuum drying at 60 °C for 12 h to obtain the NiFeS/Cs_0.32_WO_3_ composite.

### 3.5. Characterizations

The crystal structure of the samples was characterized using a Bruker D8 Advance X-ray powder diffractometer (Bruker, Bremen, Germany) with Cu Kα radiation (λ = 1.54059 Å), scanning range of 5° to 80°, and scanning rate of 5°/min. The microstructure and lattice structure of the samples were observed using a field emission transmission electron microscope (“FEI Talos F200X, Hillsboro, OR, USA). The elemental composition and chemical states of the samples were analyzed using an X-ray photoelectron spectrometer (XPS, Thermo Scientific Escalab 250Xi, Waltham, MA, USA), with all binding energies calibrated against adventitious carbon at C 1s = 284.8 eV. The optical absorption properties of the samples were measured using a UV–visible diffuse reflectance spectrometer (UV-vis DRS, Shimadzu UV-3600, Kyoto, Japan) equipped with an integrating sphere attachment, using BaSO_4_ as the reference. Photothermal conversion of the samples under near-infrared irradiation was recorded using an infrared thermal imaging camera (808 nm, 5 W).

### 3.6. Electrocatalytic Performance Testing

All electrochemical measurements were performed on a CHI660E electrochemical workstation using a standard three-electrode system, with a glassy carbon electrode (GCE, diameter 3 mm) as the working electrode, a mercury/mercury oxide electrode (Hg/HgO) as the reference electrode, a graphite rod as the counter electrode, and 1 M KOH solution as the electrolyte. Prior to formal testing, the electrodes were activated by cyclic voltammetry (CV) for 10 cycles in the potential range of 0 to 0.8 V at a scan rate of 100 mV s^−1^ to obtain stable electrochemical signals. Linear sweep voltammetry (LSV) was employed to evaluate the oxygen evolution reaction (OER) activity of the catalysts, with a potential range of 0 to 0.8 V, scan rate of 5 mV s^−1^, and reverse scanning mode. Electrochemical impedance spectroscopy (EIS) measurements were conducted at the potential corresponding to a current density of 10 mA cm^−2^, with a frequency range of 100 kHz to 1 Hz. Photoelectrocatalytic experiments employed a JH-GHX300 xenon lamp light source (Jihui Analytical Instrument Co., Shanghai, China) as the simulated light source, with an operating current of 15 A and power specifications of AC 220 V, 50 Hz. Measurements were performed under both dark and illuminated conditions; during dark measurements, the electrolytic cell was covered with a black light-blocking shield, while during illuminated measurements, the light source was directed onto the surface of the L-shaped working electrode in an optical water-bath circulating electrolytic cell, with a circulating water cooling system maintaining constant electrolyte temperature to eliminate the influence of electrolyte heating on the test results.

## 4. Conclusions

In this study, NiFeS/Cs_0.32_WO_3_ heterostructure composites were successfully prepared via a solvothermal method. Cs_0.32_WO_3_ possesses a unique hexagonal tungsten bronze structure, and the LSPR effect endows the composite with excellent near-infrared light absorption performance. Since the work function of Cs_0.32_WO_3_ (4.11 eV) is higher than that of NiFeS (3.78 eV), electrons spontaneously transfer from NiFeS to Cs_0.32_WO_3_ upon contact, forming a built-in electric field at the interface; this electron transfer behavior was confirmed by XPS, showing that the Fe 2p binding energy shifts positively by 0.72 eV after compositing, while the Cs 3d and W 4f binding energies shift negatively by 0.14 eV and 0.26 eV, respectively. The NiFeS/Cs_0.32_WO_3_-60 mg composite exhibits excellent catalytic activity in the photothermal-assisted electrocatalytic OER process, with an overpotential of only 301 mV at a current density of 50 mA cm^−2^ and a Tafel slope of 44.5 mV dec^−1^. Compared to NiFeS under dark conditions, the composite exhibits a potential reduction of 53 mV under photothermal conditions; the enhanced overall performance of the NiFeS/Cs_0.32_WO_3_ composite is primarily attributed to its photothermal enhancement effect, whereas the intrinsic interfacial effects arising from the heterojunction also contribute significantly and cannot be neglected. The performance enhancement can be attributed to the photothermal conversion effect of Cs_0.32_WO_3_ accelerating the charge transfer kinetics at the electrode/electrolyte interface, while interfacial electron transfer at the heterostructure optimizes the electronic structure of the catalyst. The heterostructure–photothermal synergistic enhancement mechanism revealed in this study can provide insights for the design of efficient electrocatalytic materials in the field of clean energy.

## Data Availability

The original contributions presented in this study are included in the article/Supplementary Materials. Further inquiries can be directed to the corresponding author.

## Supplementary Materials

The following supporting information can be downloaded at: https://www.mdpi.com/article/10.3390/molecules31132330/s1, Figure S1: EDS line scan analysis of NiFeS/Cs_0.32_WO_3_-60 mg composite: (a) TEM image showing the line scan region; (b) overview of elemental signal intensities; (c–h) line scan intensity profiles of Ni, Fe, S, Cs, W and O elements; Figure S2: Double-layer capacitance (Cdl) measurements of NiFeS and NiFeS/Cs_0.32_WO_3_ composites: CV curves of (a) NiFeS, (b) NiFeS/Cs_0.32_WO_3_-60 mg and (c) NiFeS/Cs_0.32_WO_3_-200 mg at different scan rates (20–120 mV s−1); (d) Cdl fitting curves; Figure S3: Stability test of NiFeS/Cs_0.32_WO_3_-60 mg composite; Table S1: Solution resistance (R_s_), charge-transfer resistance (R_ct_), and corresponding fitting errors for all samples.
