# Supplementary material for: Synergistic Enhancement of Electrocatalytic Oxygen Evolution via Photothermal Effect in NiFeS/Cs0.32WO3
Source: Molecules. 2026 Jul 2;31(13):2330. doi: 10.3390/molecules31132330 (PMC13362536; doi:10.3390/molecules31132330)
Supplement: Supplementary file 1 [file molecules-31-02330-s001.zip › molecules-4374682-supplementary.pdf]

Synergistic Enhancement of Electrocatalytic Oxygen Evolution via Photothermal Effect in NiFeS/$Cs_{0.32}WO_3$

Ze Wang[1,#], Xin Zhang[1,#], Wucong Wang[1], Xiong Yang[1,2], Xinyu Song[1], Shifeng Wang[1,*]

[1] Key Laboratory of Plateau Oxygen and Living Environment of Xizang Autonomous Region, and College of Science, Xizang University, Lhasa 850000, China

[2] School of Energy and Environmental Engineering, University of Science and Technology Beijing, Beijing 100000, China (yangx@ustb.edu.cn)

[#] These authors contributed equally to this work.

[*] Correspondence: wsf@utibet.edu.cn (S.W.)

Supporting Information

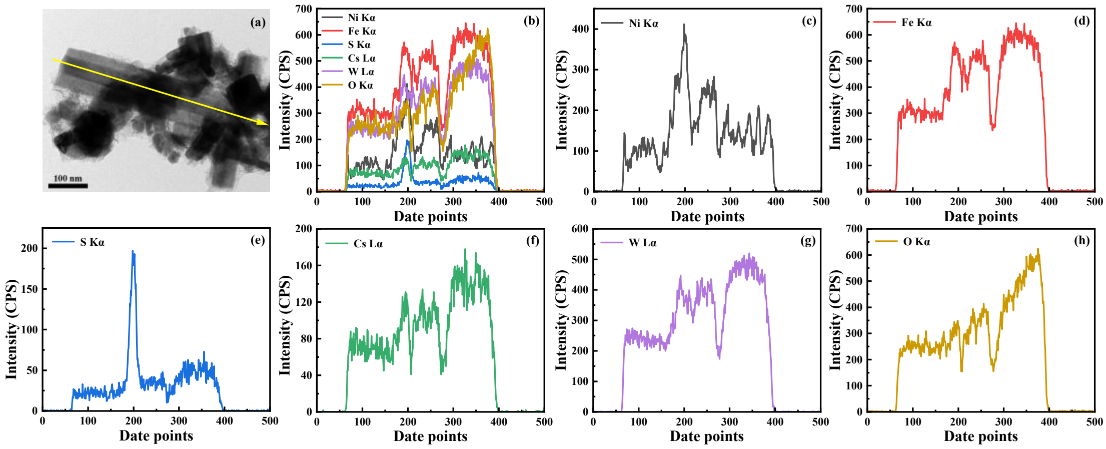

Figure S1. EDS line scan analysis of NiFeS/$Cs_{0.32}WO_3$-60 mg composite: (a) TEM image showing the line scan region; (b) overview of elemental signal intensities; (c–h) line scan intensity profiles of Ni, Fe, S, Cs, W and O elements.

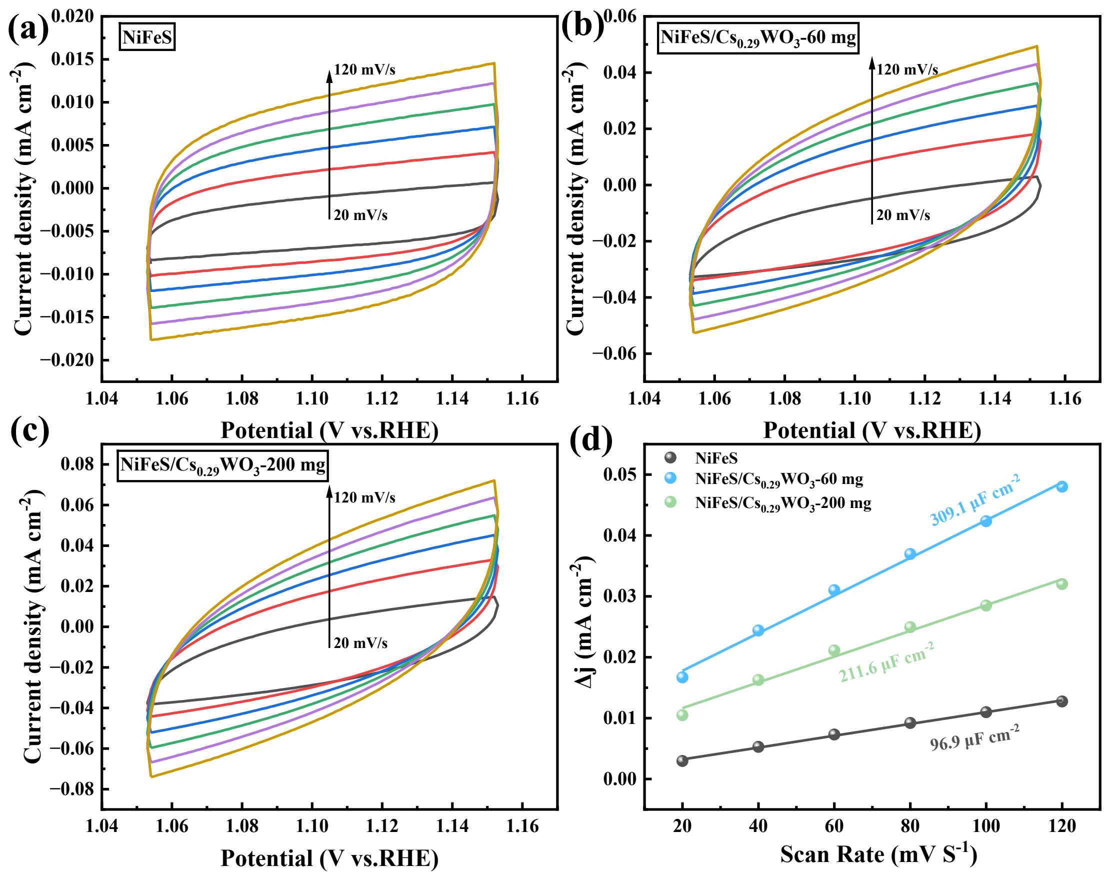

Figure S2. Double-layer capacitance (Cdl) measurements of NiFeS and $NiFeS/Cs_{0.32}WO_3$ composites: CV curves of (a) NiFeS, (b) $NiFeS/Cs_{0.32}WO_3$-60 mg and (c) $NiFeS/Cs_{0.32}WO_3$-200 mg at different scan rates (20–120 mV s^{-1}); (d) Cdl fitting curves.

Sample	Rs(Ω)	Fit error (%)	Rct(Ω)	Fit error (%)
NiFeS-Dark	8.39	0.52	10.4	3.11
NiFeS-Photothermal	8.43	0.89	6.3	10.79
NiFeS/Cs0.32WO3-60 mg-Dark	9.36	1.16	4.4	10.15
NiFeS/Cs0.32WO3-60 mg-Photothermal	9.03	0.53	3.1	14.25

Table S1. Solution resistance (R_s), charge-transfer resistance (Rct), and corresponding fitting errors for all samples.

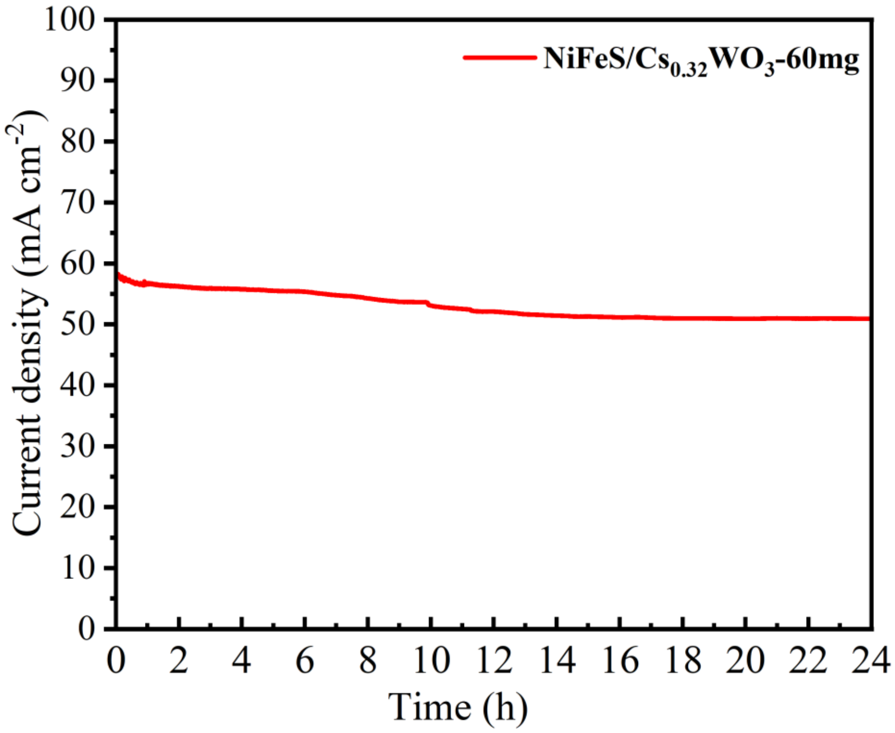

Figure S3. Stability test of NiFeS/$Cs_{0.32}WO_3$-60 mg composite
